# Writing to your past-self can make you feel better

**DOI:** 10.3389/fpsyg.2024.1327595

**Published:** 2024-02-27

**Authors:** Eriko Sugimori, Mayu Yamaguchi, Takashi Kusumi

**Affiliations:** ^1^School of Human Sciences, Faculty of Human Sciences, Waseda University, Tokyo, Japan; ^2^Division of Cognitive Psychology in Education, Graduate School of Education, Kyoto University, Kyoto, Japan

**Keywords:** autobiographical memory, depression, fading affect bias, nostalgia, group reminiscence therapy

## Abstract

Self-compassionate writing has been shown to be helpful for improving the mental state in some individuals. Here, we investigated how the writer’s attitude toward his/her past, present and future and the focus of the writing, i.e., social experience in the past versus self-experience, modulate these effects. In Experiment 1, 150 undergraduates wrote a compassionate letter to their past-self and to their future-self and responded to the Japanese version of the Adolescent Time Inventory–Time Attitudes (ATI-TA) questionnaire. Writing to past-self decreased negative feelings more than writing to future-self. Further, participants who had negative feelings toward their past, present, and future, as assessed by the ATI-TA, were more likely to be emotionally affected by writing a letter to their past-self. In Experiment 2, 31 undergraduates wrote a letter focusing on what they had experienced together with someone, and another 31 undergraduates wrote focusing on what they had experienced alone. Focusing on a social experience was more helpful for recovering from negative feelings than focusing on a self-experience. In conclusion, writing a compassionate letter to one’s past-self can improve mood, especially in individuals with a negative time attitude who focus their writing on a social connection.

## Introduction

People are likely to have a “rosy view” of their past, a phenomenon called rosy retrospection. Rosy retrospection is the tendency for an individual to evaluate past events more favorably than present events (Mitchell et al., 1997; Wirtz et al., 2003). It has been shown that pleasant life events are better recalled (Thompson et al., 2013) and come to mind more readily (Levine and Bluck, 2004) than unpleasant life events. Several studies reported that unpleasant feelings about past events fade in memory faster than pleasant feelings about past events, called the fading affect bias (Walker et al., 1997, 2003; Skowronski et al., 2014). Further, a larger emotional-intensity drop for unpleasant events than for pleasant events has been observed (Walker et al., 1997; Landau and Gunter, 2009; Ritchie et al., 2009, 2015). During a rosy view, memory is distorted in a positive way. Evidence supporting this comes from studies showing that college students remembered having more As on their high school transcripts than they actually had (Bahrick et al., 1996, 2008), and that individuals remembered their medical-test results as better than they really were (Christensen et al., 2003; Croyle et al., 2006). The rosy retrospection phenomenon is common to all cultures and has many implications for contemporary society. Above all it may serve as a coping mechanism that can promote well-being.

Our research focuses on how rosy retrospection can be harnessed to make a person feel better. In the current study, we define positive memory as a memory of a past event that elicited an emotionally positive response as described previously (Williams et al., 2022). When an individual experiences a situation with predominantly negative feelings, such negative feelings can trigger the recollection of positive memories, especially memories that are related to a social connection, which might help to overcome the challenging situation and enhance well-being. Such positive autobiographical memories act as rewards in themselves (Speer et al., 2014). Positive memories have been shown to be powerful in their ability to repair mood after a negative mood induction (Joormann and Siemer, 2004; Joormann et al., 2007). It was reported that positive memories can be used as a buffer for the effects of negative experiences (Speer and Delgado, 2017). In their study, Speer & Delgado compared the stress responses of individuals who recalled a positive or a neutral memory and showed that individuals who retrieved positive memories had a smaller cortisol response to the stressor than did individuals who retrieved neutral memories, and also reported a less negative affect. Later, the same group showed that recalling positive memories with a social component could be particularly powerful in reducing the cortisol response following the same stressor task (Speer and Delgado, 2020). That is, rosy retrospection has an impact on mental health and well-being.

Negative events can be turned into a positive memory and being listened to with compassion might be helpful in making this happen. Evidence supporting this come from studies showing that participants who elaborated on the positive aspects of past negative events reported increased positive emotions and memory content upon future recollections of the same negative event, up to 2 months after the initial recollection (Speer et al., 2021). Another study reported that memory-reframing helped children to remember a recent tonsillectomy more positively than those assigned to a control condition (Pavlova et al., 2022). Further research found that when listened to attentively, a positive memory becomes more positive, and a negative memory becomes less negative (Pasupathi and Oldroyd, 2015). It was also reported that participants who were listened to with empathy had a significantly longer talking time than non-empathy or non-response participants, higher nostalgia scores than non-response participants, and higher positive emotion scores than non-empathy and non-response participants, as well as lower negative emotion scores than non-response participants (Sugimori et al., 2020). These studies collectively show that the current feeling toward an autobiographical memory is manipulable post collection by exposure to empathy.

On the other hand, one study has reported that a substantial proportion of individuals conceal significant pieces of information and that several categories of nondisclosed information exist (Farber, 2003). In addition, some cultures (East Asia compared to Western; Chen and Danish, 2010) and some personalities (paranoid ideation; Murphy et al., 2012) are reportedly less willing to disclose themselves to an outsider. These individuals might benefit from self-compassionate interventions, such as letter writing, which can be done at one’s own discretion without disclosing private information to anyone. A lot of research has shown the positive impact of self-compassion interventions on mental health and well-being, as reviewed in Kotera and Van Gordon (2021). Previous studies have found that self-compassionate letter writing is effective in the treatment of anorexia nervosa (Kelly and Waring, 2018), in the improvement of women’s body satisfaction (Stern and Engeln, 2018), and in reducing strong shame (Swee et al., 2023) and smoking behavior (Kelly et al., 2010). Therefore, it is believed that writing a letter to one’s past-self with compassion, without having to disclose one’s past or gain empathy from others, can lead to improved well-being.

In the current study, we compared writing to past-self versus writing to future-self. Research has shown that writing to one’s future-self can have a positive impact on mental health and well-being (Chishima et al., 2021; Schippers et al., 2023). However, the mental process behind well-being achieved by writing to one’s future-self and that achieved by writing to one’s past-self might be different. According to (Chishima et al., 2021; Schippers et al., 2023), writing a letter to one’s future-self can clarify what one needs to work on to achieve one’s academic and career goals. In this sense, writing a letter to one’s future-self potentially creates anxious feelings by making oneself realize what is not sufficient for now, while writing a letter to one’s past-self comforts one’s feelings. That is, the mental states affected by writing a letter to one’s future-self and past-self would be different. In addition, the attitude toward one’s own past, present and future, which is called time attitude, would influence how the mental states change by writing a letter to one’s future and past-self. People who have a positive attitude toward their past might feel positive by writing a letter to past-self, while people who have a positive attitude toward their future might feel positive by writing a letter to future-self. In a similar way, people who have a negative attitude toward their present might be more likely to be influenced by writing to past-self because their mental state is unstable and because positive memories have an ability to repair mood after a negative mood induction (Joormann and Siemer, 2004; Joormann et al., 2007).

In the present study, we also compared writing about a social experience in the past versus writing about an event experienced alone in the past. Speer and Delgado (2020) found that people were likely to choose to reminisce about highly social memories more frequently than about less social memories of equally positive feeling and recalling memories that included higher social context led to a greater dampening of the physiological stress response (i.e., cortisol). On the other hand, Pillemer et al. (2007) found that memories of positive self-worth often focus on achievement/mastery themes, while memories of negative self-worth focus on interpersonal/affiliation themes. Under the condition of self-compassion, it would be interesting to examine whether the recall of social connections or one’s own personal memories leads to mental stability and well-being.

## Aims of the study

In the current study, we first investigated how writing a self-compassionate letter to past-self can affect the mental state in healthy individuals by comparing it to writing a self-compassionate letter to future-self in Experiment 1. We also investigated what kind of people are influenced by writing to their past−/future-selves from the viewpoint of time attitude, i.e., the attitude toward one’s own past, present and future. In Experiment 2, we investigated the effects of focusing the writing on a social experience in the past versus focusing on an event experienced alone. All participants were healthy undergraduates. Therefore, the results need to be interpreted with respect to the limited demographic characteristics of the participants. However, the findings confirmed our predictions and have implications for the development of more effective self-compassionate intervention tools.

## Methods

### Study design and study period

The study was designed as an independent measures study. Experiments were carried out from May to August 2022 (Experiment 1) and from September to October 2022 (Experiment 2).

### Participant recruitment

Participants were recruited from among undergraduate students of Waseda University by way of flyers posted around campus and by sending out recruitment e-mails. To be included in the study, participants had to be of Japanese nationality, raised in Japan, healthy, and with no history of taking psychedelic medication throughout life. Candidate participants who were currently seeking treatment for a mood or mental disorder were excluded from participation. Participation in the study was voluntary and bore no relation to course credits or performance evaluation.

### Participant informed consent

Written informed consent to participation in the study and publication of the results was obtained from all participants prior to conducting experiments.

## Measures

### Demographic characteristics

The age and sex of the participants were recorded.

### Profile of Mood States second edition short version

The Profile of Mood States second edition (POMS2) questionnaire assesses the short-term mood state, which is understood to be transient and frequently fluctuating, of individuals 13 and older. We used the paper-based Japanese short version of the POMS2, which takes about 5–10 min to complete (Yokoyama and Watanabe, 2015). The POMS2 short version contains 35 items from the full-length version of POMS2 (Heuchert and McNair, 2012). The POMS2 is composed of six subscales: Anger-Hostility (AH), Confusion-Bewilderment (CB), Depression-Dejection (DD), Fatigue-Inertia (FI), Tension-Anxiety (TA), and Vigor-Activity (VA). Only the VA subscale carries a positive valence. Items in each subscale are scored from 0 (“Not at all”) to 4 (“Extremely”) on a Likert-type scale.

### Total mood disturbance

We calculated the scores for Total Mood Disturbance (TMD) from the subscales of the POMS2 short as follows: TMD = (TA) + (FI) + (CB) + (AH) + (DD) - (VA) (Heuchert and McNair, 2012, 2015). A higher TMD score indicates a more negative mood.

### Adolescent Time Inventory–Time Attitudes

The Adolescent Time Inventory–Time Attitudes (ATI-TA) questionnaire assesses the attitude toward one’s past, present and future (Mello and Worrell, 2007; see Worrell et al., 2013) for the original ATI-TA. We used the paper-based Japanese version of the ATI-TA (Chishima et al., 2019). The ATI-TA consists of six subscales assessing two valences (positive and negative) for each of three time periods (past, present, and future). (a) Past Positive (“My past is full of happy memories”), (b) Past Negative (“My past makes me sad”), (c) Present Positive (“I am happy with my current life”), (d) Present Negative (“I am not satisfied with my life right now”), (e) Future Positive (“I am excited about my future”), and (f) Future Negative (“Thinking about my future makes me sad”). ATI-TA items are rated on a 5-point Likert-type scale from 1 (“totally disagree”) to 5 (“totally agree”).

## Procedure

All experiments were conducted at Waseda University campus in a quiet experimental room. All participants were instructed by the same experienced researcher. Before the experiment, participants were told that they will be writing a letter to their past-self and future-self with compassion. No details about the purpose or aims of the study were given. Further, to make sure they took the experiment seriously, participants were told that their letters will be collected, and the content checked by a researcher.

### Experiment 1

The experiment was an individual laboratory experiment assigned to 150 participants (54 males, 94 females, and 2 no-response; mean age = 20.86 years (SD = 2.04)). The participants sat in a chair with a desk in front of them. First, all participants were asked to answer POMS2 short. Participants were then randomly divided into two groups of 75 participants each. This was a simple randomization under the condition that the numbers of males and females are the same in the two groups. Therefore, both groups were comprised of 28 male and 47 female participants. Participants in the “past-self → future-self” group (mean age = 20.81 years, SD = 2.14) were asked to write a letter to themselves 5 years ago. Participants in the “future-self → past-self” group (mean age = 20.93 years, SD = 1.95) were asked to write a letter to themselves 5 years in the future. In both conditions, they were instructed to write in a sympathetic way as the person who understands them best. No time limit was set, but they usually finished writing within 5–10 min. After finishing the letter, they filled out POMS2 short again and they were then dismissed.

Seven days later, the participants came back and answered POMS2 short, and were then asked to write a letter to themselves 5 years in the future if they had previously written to their past-self, and to write a letter to themselves 5 years ago if they had previously written to their future-self. The instructions were the same as 7 days earlier. After finishing the letter, they answered POMS2 short again. Finally, they answered the ATI-TA. We deliberately chose a time period of 5 years for writing the letters to the past-and future-self because both represented distinct stages in the lives of the participants, i.e., high school student versus future university graduate, accompanied by characteristic environmental changes.

### Experiment 2

The experiment was an individual laboratory experiment assigned to 62 participants who had not participated in Experiment 1. The participants sat on a chair with a desk in front of them. First, participants answered the POMS2. Participants were then randomly divided into 2 groups of 31 participants each. As in Experiment 1, this was a simple randomization, therefore both groups consisted of 11 males and 20 females. Participants were asked to write a letter to themselves 5 years ago. Participants in the “self-alone” group (mean age = 20.77 years; SD = 2.01) were asked to focus their writing on something they had experienced by themselves alone. Participants in the “social connection” group (mean age = 20.13 years; SD = 1.77) were asked to focus on something they had experienced together with someone else. In both conditions, they were instructed to write in a sympathetic way as the person who understands them best. After finishing the letter, they answered POMS2 short again.

## Participant flow diagram

**Figure 1:**
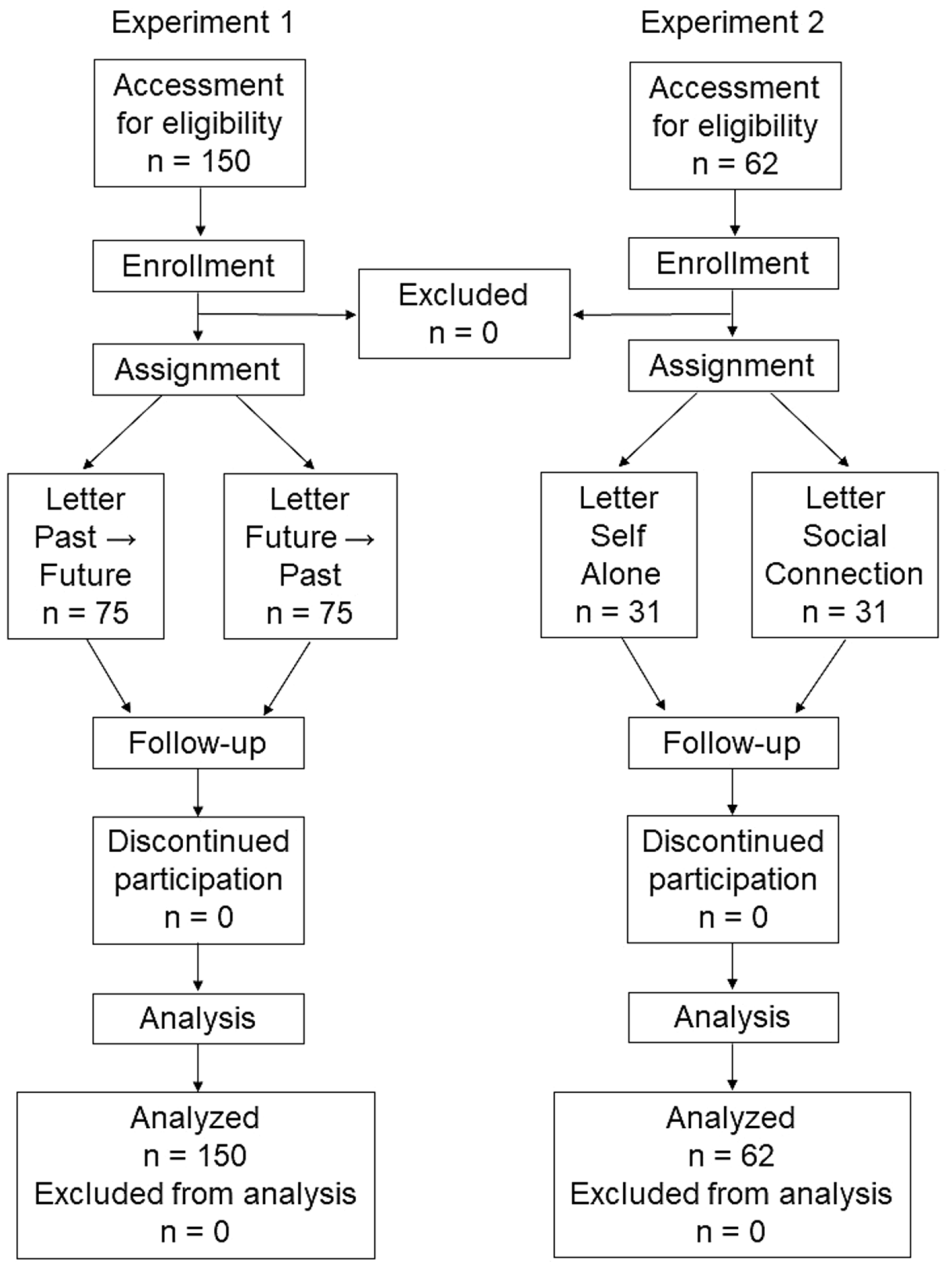
Participant flow diagrams for Experiment 1 and Experiment 2.

Participant flow diagrams for Experiment 1 and Experiment 2 are shown in Figure 1.

## Data analysis

We calculated sample size using g*power (Faul et al., 2009) under conditions of an effect size of 0.3, α of 0.05, and power (1 - β) of 0.95 in Experiment 1, and determined that the sample size in each group should be 56, while under conditions of an effect size of 0.3, α of 0.05, and power (1 - β) of 0.80 in Experiment 2, and determined that the sample size in each group should be 32.

In Experiment 1, as dependent variables based on POMS2, we used the average of [TMD (Total Mood Disturbance) score after writing the letter] minus [TMD score before writing the letter], and the averages of [each subscale (TA, FI, CB, AH, DD, VA) score after writing the letter] minus [each subscale score before writing the letter] for the writing to past-self condition and for the future-self condition to see the change of their mood by writing the letter. Differences between the two writing conditions were analyzed by analysis of variance (ANOVA), followed by the REGWQ (Ryan-Einot-Gabriel-Welsch and Quiot) post-hoc test as a multiple comparison test using JASP software. We also investigated differences in the number of people who felt positive/no change/negative after writing a letter to their past−/future-selves were analyzed using the χ2 test for whom (past-self, future-self) x mood change (positive, no change, negative), with the dependent variable being the number of people. *p*-values of less than 0.05 were considered statistically significant. Then we separated the participants in each condition (the writing to past-self condition and the writing to the future-self condition) into three groups based on their mood change after writing the letter (positive, no change, negative). The dependent variables in that case were the scores of the six subscales of the ATI-TA assessing two valences (positive and negative) for each of the three time periods (past, present, and future). We also investigated the net scores [(positive score) – (negative score)] of ATI-TA for each of the three time periods (past, present, and future) corresponding to mood changes after writing a letter to past−/future-self. Differences among those conditions were analyzed by analysis of variance (ANOVA), followed by the REGWQ (Ryan-Einot-Gabriel-Welsch and Quiot) post-hoc test as a multiple comparison test.

Using KH Coder, a quantitative text analysis software developed by Higuchi (2001), we investigated the differences in the frequency of words describing others in the letter to past-self between participants whose mood changed positively and participants whose mood changed negatively after writing the letter. Then we conducted hypothesis testing for the difference in the population proportions between the frequency of words describing others in a letter which affected the participants’ mood positively and those in a letter which affected the participants’ mood negatively.

In Experiment 2, as dependent variables, based on POMS2, we used the average of [TMD (Total Mood Disturbance) score after writing the letter] minus [TMD score before writing the letter], and the averages of [each subscale (TA, FI, CB, AH, DD, VA) score after writing the letter] minus [each subscale score before writing the letter] for the “self-alone” group and for the “social connection” group to see the mood change after writing the letter. Differences between the two writing conditions were analyzed by analysis of variance (ANOVA), followed by the REGWQ (Ryan-Einot-Gabriel-Welsch and Quiot) post-hoc test as a multiple comparison test. We also investigated the differences in the number of participants who felt positive/no change/negative after writing a letter to past-self focusing on something they had experienced by themselves versus focusing on something they had experienced together with someone else by using the χ2 test.

## Results

All data in Experiments 1 and 2 were obtained from healthy Japanese undergraduates with no history of taking psychedelic medication throughout life.

### Mood change after writing to past-self vs. after writing to future-self

In Experiment 1, we asked the participants to write a compassionate letter to their past-self and 7 days later another letter to their future-self or the other way around. In other words, we used a cross-over design to investigate how a letter to the past-self and to future-self would affect the mood in the same participant. For scoring, we calculated the change in the scores of the Total Mood Disturbance (TMD) after writing to future−/past-self as follows:

TMD change = Average of [TMD score after writing the letter] minus [TMD score before writing the letter].

A negative value for the TMD change indicates that negative feelings decreased. On the other hand, a positive value for the TMD change means that negative feelings increased.

First, we investigated if the order of letter writing had any effect on the TMD change using a two-factor ANOVA design, with “order of letter-writing” (future-self → past-self vs. past-self → future-self) × gender (female vs. male) × “to whom” (future, past). Results showed no effect of “order of letter-writing” (*F*(1, 146) = 1.00, MSE = 18.40, η2 = 0.04, *p* = 0.26) or of “gender” (*F* (1, 146) = 0.91, MSE = 16.77, η2 = 0.03, *p* = 0.20), while a main effect for the factor “to whom, past vs. present” was observed (*F*(1, 148) = 60.21, MSE = 8.51, η2 = 0.64, *p* < 0.01). Therefore, the factors of “order of writing letter” and “gender” were discarded.

The results in Table 1 show that the number of participants who had a more positive mood after writing a letter to past-self was larger than that of participants who had a more positive mood after writing a letter to future-self. In addition, the number of participants who had a more negative mood after writing a letter to future-self was larger than that of participants who had a more positive mood after writing a letter to past-self (x2 (2) = 29.921, *p* < 0.01).

**Table 1:** Number of participants by mood change after writing a letter to past- or future-self.

	More positive mood	No change	More negative mood
Letter to past-self (*n*)	88	18	44
Letter to future-self (*n*)	44	17	89

**Figure 2:**
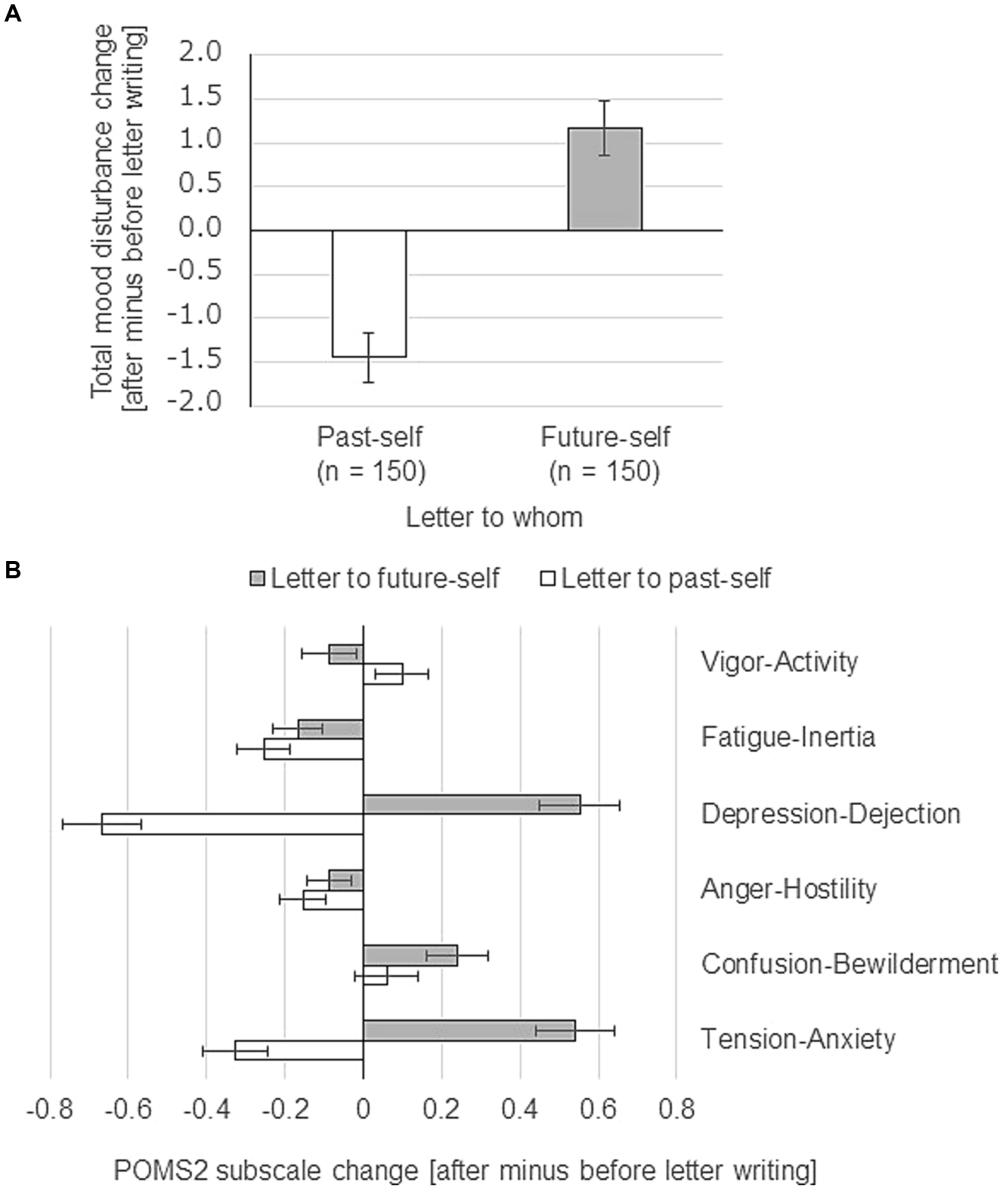
Mood change after writing a letter to past-self or future-self. Participants were divided into two groups of 75 participants each. One group wrote a compassionate letter first to their past-self and 1 week later to their future-self. The other group wrote a compassionate letter first to their future-self and 1 week later to their past-self. POMS2 was answered before and after the letter writing. Total Mood Disturbance (TMD) was calculated from the POMS2 subscale scores. **(A)** Changes in TMD after writing the letter. **(B)** Contribution of POMS2 subscales to the change in TMD shown in panel **(A)**. Data shown are mean ± SEM.

Next, we investigated the magnitude of change in mean TMD (TMD after writing the letter minus TMD before writing the letter). As can be seen from Figure 2A, writing a letter to past-self led to a negative TMD change, meaning the mood increased. In contrast, writing a letter to future-self led to a positive TMD change, meaning the mood decreased. To investigate how each of the POMS2 subscales contributed to the overall TMD change, we conducted a 2 (past vs. future) × 6 (TA, FI, CB, AH, DD, VA) ANOVA on the data ([after minus before] subscale change). The interaction was significant. Subscale changes were significantly lower when writing to past-self than when writing to future-self for the subscales TA (*F*(1, 149) = 80.19, *p* < 0.01), DD (F(1, 149) = 118.92, *p* < 0.01), and CB (F(1, 149) = 5.40, *p* < 0.05) (Figure 2B). The subscale change was significantly higher when writing to past-self than when writing to future-self for the subscale VA (F(1, 149) = 5.87, *p* < 0.05) (Figure 2B). No significant difference in subscale change was found for AH (F(1, 149) = 1.48, ns) or FI (F(1, 149) = 1.62, ns) (Figure 2B). These results show that reduced TA and DD scores and an increased VA score were the main drivers of the increase in mood after writing a letter to past-self.

### Average scores of ATI-TA correspond to mood changes after writing letters to past-self

**Figure 3:**
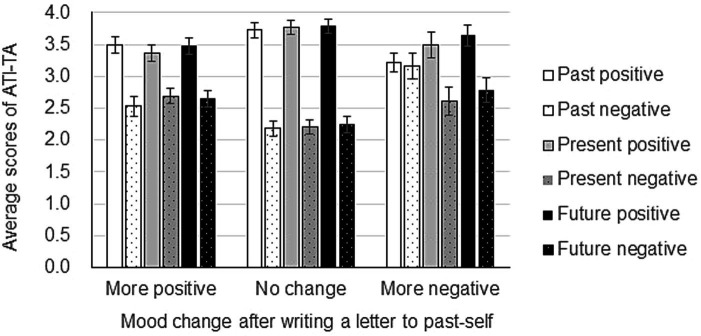
Mood change after writing a letter to past-self depending on time attitude. One hundred and fifty participants wrote a compassionate letter to their past-self. POMS2 was answered before and after the letter writing and the change in total mood disturbance was calculated from the POMS2 subscale scores. In the end, all participants answered the Adolescent Time Inventory–Time Attitudes (ATI-TA) questionnaire. Data shown are mean ± SEM.

To further investigate what kind of participants are influenced by writing to their past-self, we employed the ATI-AT scale to measure the participants’ attitude toward their past, present, and future. Then, we investigated how average scores of ATI-TA correspond to mood changes after writing letters to past-self using ANOVA, with mood after writing a letter (between: more positive, no change, more negative) X time (within: past, present, future) X mood score (within: positive, negative). Results showed that the main effect of mood after writing a letter was significant (*F*(2, 147) = 4.78, MSE = 0.36, *p* < 0.05, no change < more negative). Further, the main effect of mood score was also significant (*F*(1, 147) = 67.15, MSE = 2.69, *p* < 0.01, negative scores < positive scores). In addition, the interaction between mood after writing a letter X mood score was significant (F(2, 147) = 5.38, MSE = 2.69, *p* < 0.01). Regarding a positive score, there was no difference in mood after writing the letter (*F*(2, 294) = 2.48, MSE = 1.53, *p* = 0.06). Regarding a negative score, there was a significant mood difference after writing the letter (F(2, 294) = 8.12, MSE = 1.53, *p* < 0.01, no change < more positive, no change < more negative) (Figure 3). Together, these findings indicate that participants who have a less negative attitude toward time were not affected by writing a letter to their past-self, neither positively nor negatively.

### The net score [(positive score) – (negative score)] of ATI-TA corresponds to mood changes after writing a letter to past-self

**Figure 4:**
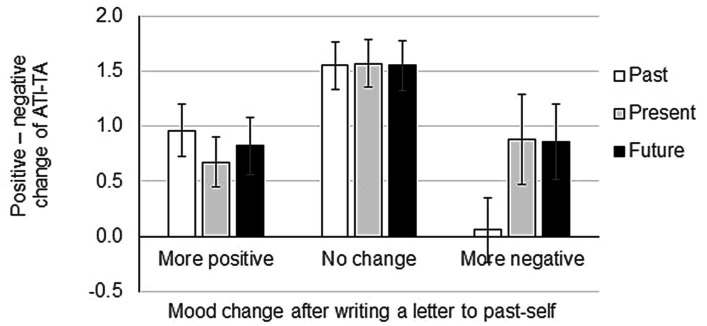
Mood change after writing a letter to past-self in relation to the Adolescent Time Inventory–Time Attitudes (ATI-AT) net score. POMS2 was answered before and after the letter writing and the total mood disturbance was calculated from the POMS2 subscale scores. Participants answered the ATI-TA questionnaire after finishing writing a compassionate letter to past-self. Net scores (positive minus negative) of the ATI-TA are indicated on the Y-axis. Data shown are mean ± SEM.

After revealing that the time attitude of an individual affects the mood change after writing a compassionate letter to past-self, we next wanted to investigate how the net score of ATI-AT corresponds to mood changes after writing letters to past-self. We first calculated the net score of ATI-AT by subtracting the score for the negative time attitude subscales from the score for the positive time attitude subscales. Then, we submitted the ATI-AT net score to an ANOVA with mood after writing a letter (between: more positive, no change, more negative) X time (within: past, present, future). Results showed that the main effect of mood after writing a letter was significant (*F*(2,147) = 28.99, MSE = 5.39, *p* < 0.01, no change > more negative, no change > more positive). However, the main effect of mood score was not significant (*F*(2,294) = 1.94, MSE = 2.16, *p* = 0.90). Similarly, the interaction between mood after writing a letter X mood score was not significant (*F*(4,294) = 3.81, MSE = 2.16, *p* = 1.76) (Figure 4). These results indicate that although participants who became more negative after writing a letter to past-self scored almost zero on their past attitude in the ATI-AT score, this did not reach statistical significance.

### Average scores of ATI-TA correspond to mood changes after writing letters to future-self

**Figure 5:**
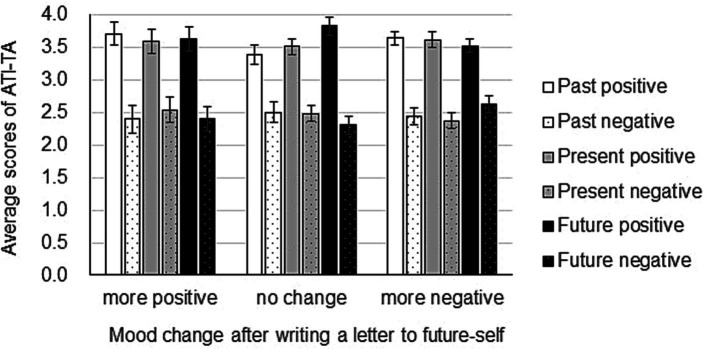
Mood change after writing a letter to future-self depending on time attitude. One hundred and fifty participants wrote a compassionate letter to their future-self. POMS2 was answered before and after the letter writing and the change in total mood disturbance was calculated from the POMS2 subscale scores. At the end, all participants answered the Adolescent Time Inventory–Time Attitudes (ATI-TA) questionnaire. Data shown are mean ± SEM.

Next, we investigated how the mean scores of ATI-TA correspond to mood changes after writing a letter to future-self. ANOVA using the factors mood after writing a letter (between: more positive, no change, more negative) X time (within: past, present, future) X mood score (within: positive, negative) showed that the main effect of mood after writing a letter was not significant (*F*(2, 147) = 0.14, MSE = 0.38, ns). In addition, the main effect of mood score was significant (*F*(1, 147) = 250.39, MSE = 3.02, *p* < 0.01, negative scores < positive scores). Further, the interaction between mood after writing a letter X mood score was significant (F(2, 147) = 0.13, MSE = 3.02, *p* = 0.04) (Figure 5). These findings show that a participant’s attitude toward time affects the mood after writing a letter to future-self.

### The net score [(positive score) – (negative score)] of ATI-TA does not correspond to mood changes after writing a letter to future-self

**Figure 6:**
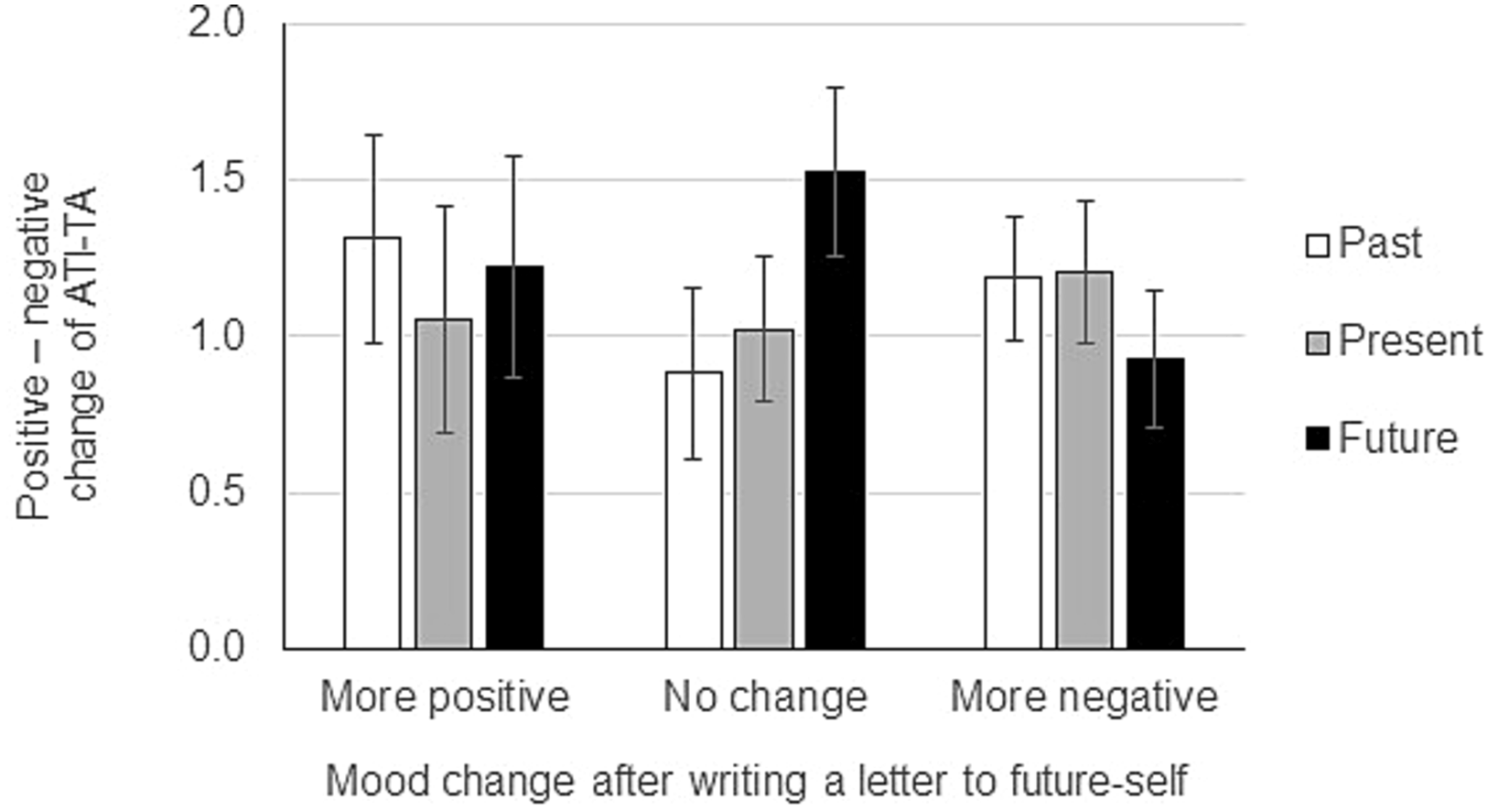
Mood change after writing a letter to future-self in relation to the Adolescent Time Inventory–Time Attitudes (ATI-AT) net score. POMS2 was answered before and after the letter writing and the total mood disturbance was calculated from the POMS2 subscale scores. Participants answered the ATI-TA questionnaire after finishing writing a compassionate letter to future-self. Net scores (positive minus negative) of the ATI-TA are indicated on the Y-axis. Data shown are mean ± SEM.

After revealing that the time attitude of an individual affects the mood change after writing a compassionate letter to future-self, we next wanted to investigate how the net score of ATI-AT corresponds to mood changes after writing a letter to future-self. We first calculated the net score of ATI-AT by subtracting the score for the negative time attitude subscales from the score for the positive time attitude subscales. Then, we submitted the ATI-AT net score to an ANOVA with mood after writing a letter (between: more positive, no change, more negative) X time (within: past, present, future). Result showed that the main effect of mood after writing a letter to future-self was not significant (*F*(2,147) = 0.04, MSE = 6.04, ns). The main effect of mood score was also not significant (*F*(2,294) = 0.29, MSE = 2.11, ns). Finally, the interaction between mood after writing a letter to future-self X mood score was not significant (*F*(4,294) = 1.41, MSE = 2.11, ns) (Figure 6).

### Differences in the frequency of words describing others in the letter to past-self when the mood changed positively versus when the mood changed negatively after writing the letter

Using KH Coder, a quantitative text analysis software developed by Higuchi (2001), we extracted all the written words from the text data of letters to past-selves, and examined the differences in the number of words describing others in the letters by participants whose mood changed positively versus participants whose mood changed negatively after writing the letter. Words regarded as used to describe others were: friends, teachers, classmates, peers, family, parents, father, mother, people around them, seniors, juniors, etc. In the letters which created a positive mood, there were 63 words (0.72 words per person) describing others, and in the letters which created a negative mood, there were 21 words (0.48 words per person) describing others. Hypothesis testing for the difference in the population proportions revealed that the proportion of words describing others in the letter of participants whose mood changed positively after writing a letter was significantly higher than the proportion of words describing others in the letter of participants whose mood changed negatively after writing the letter (*α* = 0.05, *g* = 0.0996, *p* = 0.0125).

### The effects of focusing the writing on a social experience in the past versus focusing on an event experienced alone

**Figure 7:**
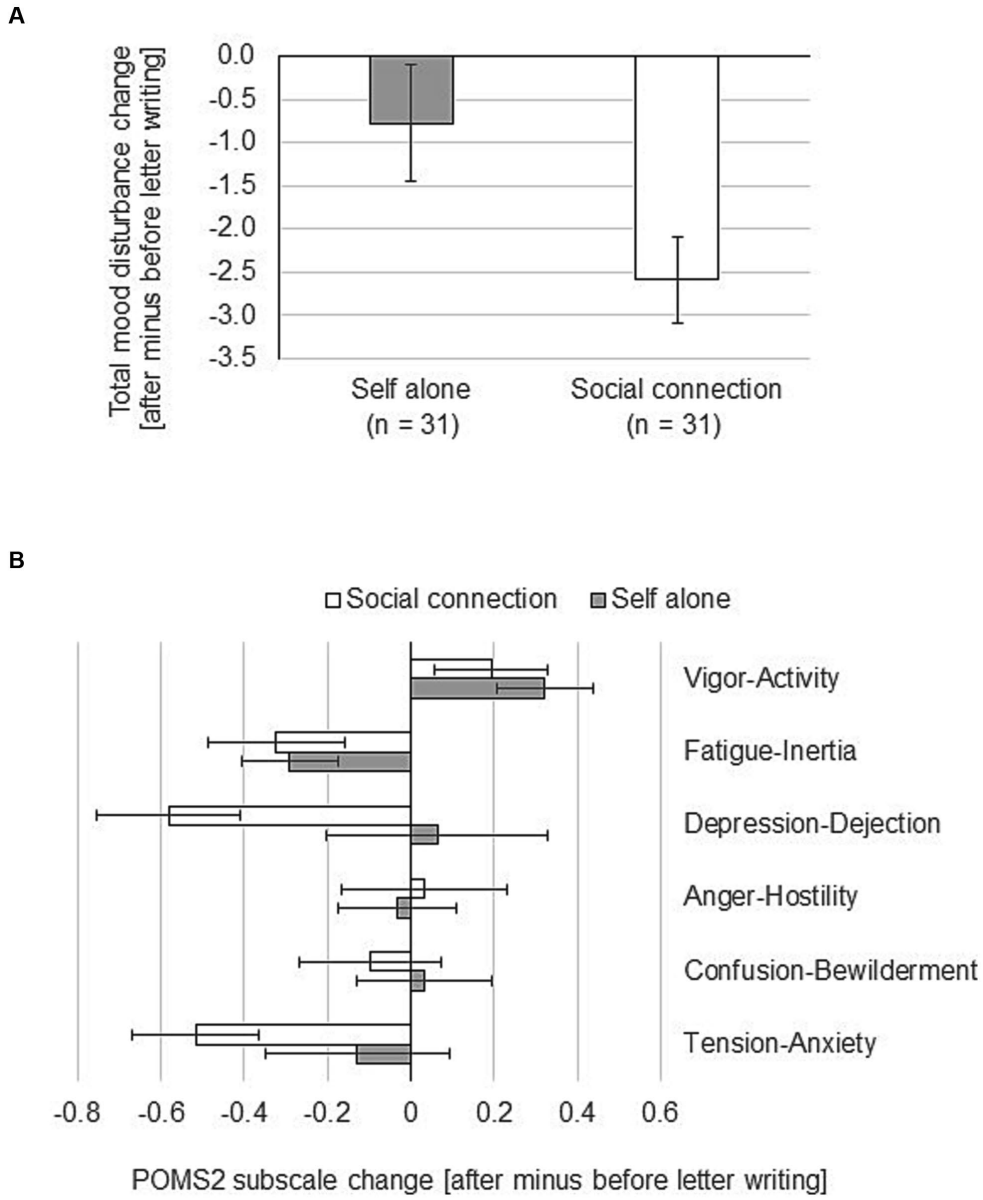
Mood change after writing a compassionate letter to past-self with a focus on a self-alone experience or on a social connection experience. Participants were divided into two groups of 31 participants each. One group wrote with a focus on a self-alone experience. The other group wrote with a focus on a social connection experience. POMS2 was answered before and after the letter writing. Total Mood Disturbance (TMD) was calculated from the POMS2 subscale scores. **(A)** Changes in TMD after writing the letter. **(B)** Contribution of POMS2 subscales to the change in TMD shown in panel **(A)**. Data shown are mean ± SEM.

In Experiment 2, we wanted to see how writing about a social connection versus writing about a self-experience affects mood. Therefore, we asked the participants in the “self-alone” group to focus their writing on something they had experienced by themselves alone and participants in the “social connection” group to focus on something they had experienced together with someone else. For scoring, we calculated the change in TMD scores after writing to past-self in the same way as in Experiment 1. ANOVA with “TMD after writing to past-self” X “focus of writing” (self vs. social connection) showed that there was a significant main effect for “focus of writing” (*F*(1, 60) = 4.81, MSE = 10.51, *f* = 0.28, *p* < 0.05; x2(2) = 13.549, *p* < 0.01) (Table 2; Figure 7A, respectively). These results show that when participants focused their writing on what they did with someone else, they became less negative than when they focused on what they experienced by themselves alone. To investigate how the POMS2 subscales contributed to this mood change, we conducted a 2 (between self-alone vs. social connection) × 6 (within TA, FI, CB, AH, DD, VA) ANOVA on the data ([after minus before] for each subscale of POMS2). The interaction was not significant (*F*(5, 300) = 1.36, MSE =0.78, η2 = 0.15, ns). The main effect of the factor of the way of letter was not significant (F(1, 60) = 2.42, MSE = 1.69, η2 = 0.20, ns). The main effect of the factor of subscales was significant (F(1, 60) = 4.13, MSE = 0.78, η2 = 0.26, *p* < 0.01), with VA > FI, VA > DD, and VA > TA (Figure 7B). The DD subscale score in the social connection group decreased significantly more than the DD subscale score in the self-alone group (*t* test, *p* < 0.05).

**Table 2:** Number of participants by mood change after writing a letter to past-self.

Focus of writing	More positive mood	No mood change	More negative mood
Self-alone	15	5	11
Social connection	28	2	1

## Discussion

In this study we investigated how writing a compassionate letter to oneself can make us feel better. The findings of Experiment 1 showed that writing a letter to past-self is effective in decreasing negative mood and writing a letter to future-self is effective in increasing negative mood. This is in line with a previous report showing that writing to the future-self can reduce delinquency (van Gelder et al., 2013; Rutchick et al., 2018). Connecting the current-self to the future-self might induce anxiety about the future, which in turn prevents the current self from doing something bad, or alternatively might promote positive future-oriented behaviors, such as self-control, money saving, and academic performance. In contrast, a study conducted in the early stage of the COVID-19 pandemic found that writing to future-self led to a decrease in negative affect and an increase in positive affect (Chishima et al., 2021). This could be explained as follows. When people are in a stressful situation, and when they know the bad situation (i.e., Covid-19 pandemic) will definitely disappear at some point in the future, writing to future-self is effective in improving the mental state. In contrast, when people are uncertain if the stressful situation will go away, writing to future-self might not comfort one’s mental state.

Regarding the time attitude, Experiment 1 also showed that people who have a less negative attitude toward their past, present, and future are less likely to be emotionally affected by writing letters to their past-self. That is, people who are content with their past, present, and future would not be mentally affected by writing a letter to their past-self. In addition, people whose mood changed negatively after writing a letter to past-self were not positive toward their past. The results of Experiment 1 are consistent with previous studies which suggested that negative feelings in a current situation can trigger positive memory, especially memory related to a social connection, which might enhance well-being (Joormann and Siemer, 2004; Joormann et al., 2007). This time, we found that some participants did not have rosy memories, although they were fewer than those who did, and for these participants writing a letter to past-self may not lead to mental health and well-being.

Qualitative text analysis in Experiment 1 showed that the number of words regarded as describing others in the letter to past-self was greater for participants whose mood changed positively compared to participants whose mood changed negatively after writing the letter. The results of Experiment 2 also showed that when participants focused their self-compassionate letter on what they did with someone else, they became less negative than when they focused on what they experienced by themselves alone. The difference in TMD change was substantial, showing that remembering the feeling of being connected with other people is highly effective in overcoming a negative mood compared to remembering something one did alone. Interestingly, the TMD change for “past-self” in Experiment 1 (Figure 2) amounts to about the average of the TMD change from “self-alone” and “social connection” in Experiment 2 (Figure 7A), most likely reflecting this mixture in the focus of the writing. We then looked further into changes of POMS2 subscales in Experiment 2 and found that the DD subscale score in the social connection group decreased significantly more than the DD subscale score in the self-alone group, which contributed to the mood increase seen in the social connection group. Therefore, a lack of social connection and/or a feeling of loneliness when writing the letter likely resulted in a more negative mood in the self-alone group. This is in agreement with a previous report showing that recalling positive memories with a social component could be particularly powerful in reducing the cortisol response following the same stressor task (Speer and Delgado, 2020) and a meta review about long-term care residents which concluded that good social connection is linked to better mental health outcomes (Bethell et al., 2021). On the other hand, some previous studies showed that achievement themes were prominently represented in memories of positive self-regard and interpersonal themes were prominently represented in memories of negative self-regard (Pillemer et al., 2007, 2013). Considering those findings, it is reasonable that remembering interpersonal problems might be more depressing than anything else, whereas thinking about your achievements with lots of support from your surroundings might be more comforting than anything else.

The current results need to be interpreted within the limits of this study and considering the narrow demographic characteristics of the participants. However, previous studies showed that writing about their memories by themselves has benefits for older people’s well-being, and that writing about memories might have a therapeutic effect (Botella and Feixas, 1993; Elford et al., 2005). Robinson and Murphy-Nugen (2018) also showed the benefits of life review writing for older adults who are at risk of isolation and depression. Johnson and O’Brien (2013) and Urken and LeCroy (2021) also found that self-compassionate writing interventions led to improvements in self-compassion and mental health. Therefore, writing letters to one’s past-self appears to be a good method for people of various ages and with a wide range of mental health conditions. Because Pauley and McPherson (2010) noted that individuals with depression or anxiety might find it challenging to practice self-compassion, suggesting the need for tailored interventions, compassionate letter writing to self might require additional support or professional guidance for people with these conditions. Future studies should therefore investigate a variety of age groups and clinically relevant study populations. In addition, there might be potentially confounding variables or factors that could have influenced the observed mood changes seen in the present study. Factors like the participants’ current life circumstances, mental health conditions, or other external events could have potentially confounded the results. A more thorough exploration of these variables will strengthen the interpretation of future findings. Another limitation is that the participant number in Experiment 2 was slightly smaller than expected. In the future, it should be of interest to investigate if writing to past-self could be used as a regular treatment to improve mood and relief depression. Similarly, it would be interesting to study if writing a letter to future-self on a regular basis can be effective in attaining measurable improvements in future behavior, performance and success in life.

## Conclusion

This study revealed that writing a letter to your past-self with compassion can be effective in transiently elevating one’s mood, especially when the focus is on an experience with friends, family, or acquaintances. When there are no negative feelings in a present situation, writing a letter to past-self will not affect mood. Further, writing a letter to your future-self can be effective in decreasing mood, which might be useful to induce motivation for self-improvement in the future. Together, these findings show how writing to past-self and future-self can effectively alter one’s mood, which has implications for the future design of self-compassionate intervention tools and their application in everyday life and in therapeutic settings.

## Data availability statement

The raw data supporting the conclusions of this article will be made available by the authors, without undue reservation.

## Ethics statement

The studies involving humans were approved by Waseda Ethics Review Procedures concerning Research with Human Subjects (approval No. 2021-163). The studies were conducted in accordance with the local legislation and institutional requirements. The participants provided their written informed consent to participate in this study.

## Author contributions

ES: Conceptualization, Data curation, Formal analysis, Investigation, Writing – original draft, Writing – review & editing. MY: Formal analysis, Investigation, Writing-review & editing. TK: Funding acquisition, Supervision, Writing – review & editing.
